# Setting Up a “Green” Extraction Protocol for Bioactive Compounds in Buckwheat Husk

**DOI:** 10.3390/ijms26157407

**Published:** 2025-07-31

**Authors:** Anna R. Speranza, Francesca G. Ghidotti, Alberto Barbiroli, Alessio Scarafoni, Sara Limbo, Stefania Iametti

**Affiliations:** Department of Food, Environmental and Nutritional Sciences, University of Milan, 20133 Milan, Italy; anna.speranza@unimi.it (A.R.S.); francesca.ghidotti@unimi.it (F.G.G.); alessio.scarafoni@unimi.it (A.S.); sara.limbo@unimi.it (S.L.); stefania.iametti@unimi.it (S.I.)

**Keywords:** extraction, polyphenols, microwave-assisted extraction, ultrasound-assisted extraction, buckwheat husk, anti-inflammatory properties

## Abstract

Buckwheat, a gluten-free pseudocereal, is rich in dietary fiber, minerals, high-quality proteins, vitamins, and essential amino acids. Buckwheat husk, a by-product of dehulling, contains high levels of bioactive compounds such as polyphenols and dietary fibers. This study compares green extraction methods (ultrasound-assisted extraction, UAE; and microwave-assisted extraction, MAE) for recovering polyphenols from buckwheat husk. MAE improved polyphenol yield by 43.6% compared to conventional acidified methanol extraction. Structural and chemical analyses of the residual husk material using SEM, FTIR, and fiber analysis revealed that MAE alters husk properties, enhancing polyphenol accessibility. Thus, MAE appears an efficient and sustainable alternative to acid- and solvent-based extraction techniques. Extracts obtained via “green” methods retained strong antioxidant activity and showed significant modulation of inflammatory markers in human Caco-2 cells, highlighting the potential use of “green” buckwheat husk extracts for food and pharma applications. This work supports the valorization of buckwheat husk within a circular economy framework, promoting buckwheat husk as a valuable raw material for bioactive compound recovery in diverse applications.

## 1. Introduction

Buckwheat is a dicotyledonous pseudocereal belonging to the Polygonaceae family. Although it is not a cereal, it is similar to cereals regarding cultivation and usage. Two buckwheat species are primarily cultivated: common buckwheat (*Fagopyrum esculentum*) and tartary buckwheat (*Fagopyrum tartaricum*). Buckwheat has garnered considerable attention for its high nutritional value, being rich in dietary fiber, resistant starch, rutin, D-chiro-inositol, vitamins, minerals, and high-quality proteins [1]. Notably, the low prolamin content and the absence of protein sequences toxic for celiacs make buckwheat a suitable starting material for gluten-free foods, addressing the dietary needs of celiac individuals or of people with gluten-related disorders [2].

The initial step in buckwheat processing is decortication, which generates a significant amount of solid waste in the form of buckwheat husk. The FAO estimates annual global buckwheat production of approximately 2.2 million tons in the year 2023 [3], with husk by-products accounting for 30–40% of the processed material [4]. One of the key objectives of the European Green Deal is the sustainable utilization of food by-products and waste. Despite being a valuable source of primary and secondary nutrients, including essential micronutrients, buckwheat husk is currently used primarily as fuel and, to a small extent, as a filler for therapeutic pillows and mattresses [5]. Among the different parts of the buckwheat plant, the hull contains the highest levels of non-starch carbohydrates (approximately 90%) and polyphenols, demonstrating superior antioxidant activity compared to the whole seed and to de-hulled grains [6,7,8]. The polyphenol content in buckwheat husk is 852 mg/kg; rutin (130 mg/kg) and vitexin (140 mg/kg) represent the two main polyphenols [9], but more than 60 phenolic compounds have been identified in the extracts [7]. Polyphenols extracted from buckwheat husk have been shown to confer various health benefits, including antidiabetic, antioxidant, anticancer, and anti-inflammatory properties, as well as protection against oxidative damage and lipid accumulation [10,11,12]. These bioactive compounds hold significant potential for industrial applications, including their use as natural flavoring agents and as food preservatives [13].

The recovery of bioactive substances from buckwheat husk highly depends on the extraction methods employed. Typically, polyphenols are extracted using techniques such as solvent extraction, digestion, percolation, and maceration. All these conventional methods present several limitations, including prolonged extraction times, high energy consumption, potential degradation of polyphenols, and excessive solvent usage. These drawbacks have driven research toward developing innovative extraction technologies that are more efficient, with lower solvent and energy consumption and the ability to preserve polyphenols’ bioactivities.

Among the “green” non-conventional extraction methods, the most well-known are ultrasound-assisted extraction (UAE) and microwave-assisted extraction (MAE). UAE utilizes ultrasonic waves to agitate a sample immersed in a biological solvent, relying on the phenomenon of cavitation. During this process, bubbles generated by the ultrasound amplitude expand over time until they reach a critical threshold, with a localized increase in temperature and pressure within the solids. This intense energy disrupts cell walls, enhancing the efficiency of extracting bioactive compounds [14]. MAE, on the other hand, relies on the use of microwave energy to heat solvents quickly and evenly. The internal temperature of the sample matrix is increased by rapid heating, which often exceeds the solvent’s boiling point in a closed system, disrupting the cell walls and membranes, thereby enhancing the solubility and diffusion of target compounds into the solvent [15]. Several studies have demonstrated the effectiveness of UAE and MAE in recovering bioactive compounds from various food by-products, such as pomegranate peels, olive pomace, and grape pomace [16,17,18]. These techniques offer significant advantages, including decreased organic solvent consumption, short extraction times, high selectivity, and improved extraction yields, making them sustainable and efficient approaches for valorizing agricultural waste [14]. Given these considerations, in this study we applied UAE and/or MAE to extract phenolic compounds from buckwheat husk, performing also a coarse characterization of the residual solid material to verify both efficiency and sustainability.

## 2. Results

### 2.1. Profiling Bioactive Compounds in Various Extracts

The “green” extraction techniques were evaluated in comparison to (i) the conventional solvent extraction based on the use of acidified methanol (Met-HCl) and (ii) the extract obtained using solely acidified water (Aqueous Acetic Acid Extract, BA), i.e., the same solvent used in MAE and UAE, to evaluate the chemical contribution in the extraction excluding the effects of physical processes. (Table 1). UAE did not result in an enhancement in the extraction of polyphenolic compounds; the quantified amount was comparable to that obtained using solely acidified water (BA = 1.05 ± 0.01 mg GAE/g, UAE = 1.11 ± 0.02 mg GAE/g). Although numerous studies in the literature have shown that UAE is highly efficient for extracting polyphenols, we did not observe this result in our study. This discrepancy is likely due to the use of milder extraction conditions compared to those typically reported, where frequencies of at least 20 kHz are typically applied [19,20,21,22,23,24]. Key parameters such as temperature, extraction time, ultrasonic power, frequency, and the type of solvent significantly influence extraction efficiency. In our case, the less intense settings may have resulted in yields of polyphenols not significantly different from BA. The wooden matrix of buckwheat husks may also represent another factor that limits the amount of extracted polyphenols [25]. Among the green extraction methods, MAE resulted in the highest polyphenol yield. Specifically, MAE was applied in two steps where after a first extraction step (MAE1), the solid residue was subjected to a second extraction step (MAE2). The best yields were obtained in MAE2, with a 43.6% increase in polyphenols compared to the conventional extraction method. The efficacy of MAE could be related to the fact that microwaves induce dipole rotation in the lignocellulosic molecules, leading to a breakdown of the structure and facilitating penetration of the solvent and solubilization of the molecules of interest [25,26].

The antioxidant capacity, expressed as mmol of Trolox equivalents (TE), was evaluated in relation to the total phenolic content (TPC) for the different extraction methods (Table 1). According to the ABTS assay, the extract obtained with MAE1 showed the highest antioxidant content per gram of TPC, followed by MAE2 and Met-HCl, which did not differ significantly from each other. Despite the low TPC observed in BA and UAE extracts, their normalized antioxidant activities remained high, with only a minor decrease with respect to Met-HCl. The DPPH assay showed a different trend. The highest antioxidant activity was observed for Met-HCl extracts, followed by MAE2 and UAE and by the least efficient BA and MAE1. The differences between ABTS and DPPH data can be attributable to the varying reactivity of some polyphenol groups when using the DPPH test, which reportedly may underestimate the antioxidant activity of species that are slowly reacting with the DPPH radical [27].

The discrepancy between the TPC values and antioxidant capacities of UAE and MAE extracts could be explained by a detrimental effect of microwaves on antioxidant activity. This was observed by Hanula et al. [19], who reported that although similar TPC levels were obtained from açai berry polyphenol extraction using both ultrasound-assisted and microwave-assisted methods, the ultrasound extract exhibited significantly higher antioxidant activity compared to the microwave extract.

**Figure 1:**
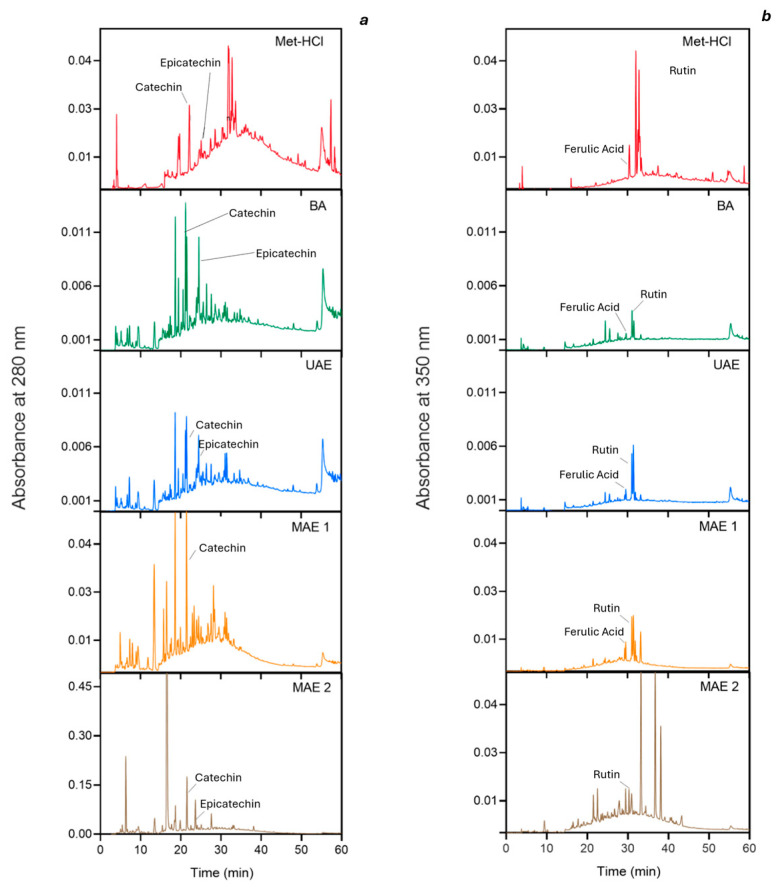
Chromatograms of the extracts recorded at two different wavelengths: (**a**) 280 nm and (**b**) 350 nm. Met-HCl, Acidified methanol extracts; BA, Aqueous Acetic Acid Extract; UAE, Ultrasound-assisted extraction; MAE1, Microwave-assisted extraction first step; MAE2, Microwave-assisted extraction second step.

The profile of phenolics in the various extracts is presented in Figure 1. Two distinct wavelengths were used for detection of individual species: 280 nm for catechin and epicatechin, and 350 nm for ferulic acid, rutin, and quercetin. The extracts obtained through “green” extraction methodologies exhibited distinct profiles compared to those derived from the conventional Met-HCl extraction, if not for UAE. UAE had the lowest impact on the phenolic profile, which appeared to be consistent with that obtained by using the standard Met-HCl extraction method. In contrast, MAE treatments resulted in significantly altered phenolic profiles, particularly evident in subsequent extraction steps.

### 2.2. Biological Activity

**Figure 2:**
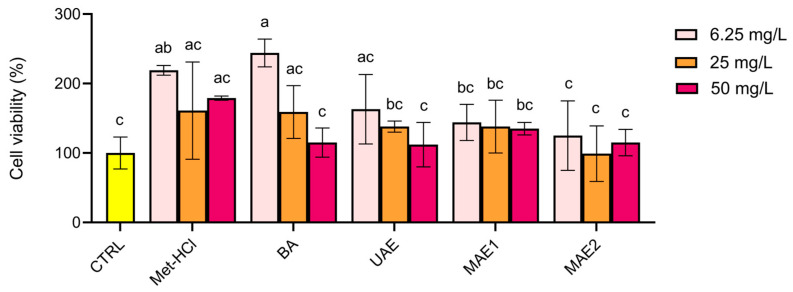
Cell viability at three different concentrations of the various extracts. The cell viability was calculated by comparing the optical density at 565 nm of treated cells to that of the control group (CTRL). Met-HCl, Acidified methanol extract; BA, Aqueous Acetic Acid Extract; UAE, Ultrasound-assisted extraction; MAE1, Microwave-assisted extraction first step; MAE2, Microwave-assisted extraction second step. Results are presented as means ± SD (*n* = 4). Data marked with the same letter are not significantly different (*p* < 0.05, Tukey’s test).

The cytotoxicity of the extracts was assessed by using the MTT assay on Caco-2 cells at total phenolic concentrations of 6.25, 25, and 50 mg/L (Figure 2). None of the extracts exhibited cytotoxic effects, as cell viability remained above 100% in all treated groups. Notably, exposure to the lower concentrations (6.25 and 25 mg/L) resulted in a slight increase in cell viability compared to untreated control. This suggests a possible proliferative effect—as opposed to cytotoxicity—at these concentrations.

**Figure 3:**
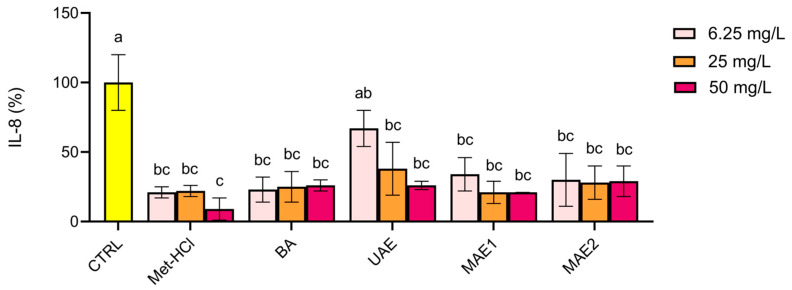
Anti-inflammatory properties of the various extracts at three different concentrations. Cells were then stimulated with IL-1β 20 ng/mL (CTRL) in the presence or absence of buckwheat extracts. IL-8 gene expression was quantified using qPCR and normalized to CTRL. Met-HCl, Acidified methanol extract; BA, Aqueous Acetic Acid Extract; UAE, Ultrasound-assisted extraction; MAE1, Microwave-assisted extraction first step; MAE2, Microwave-assisted extraction second step. Results are presented as means ± SD (*n* = 2). Data marked with the same letter are not significantly different (*p* < 0.05, Tukey’s test).

The anti-inflammatory effects of the extracts were assessed by measuring the IL-8 gene expression in IL-1β-stimulated Caco-2 cells using RT-PCR (Figure 3). The results indicated that all concentrations of the tested extracts (6.25, 25, and 50 mg/L) significantly decreased IL-8 expression compared to the IL-1β-induced control group (100% expression of IL-8). This indicates that the extracts show potential anti-inflammatory properties. These findings were consistent with the profile of the conventional extraction method using Met-HCl and represent a further support of the anti-inflammatory potential of the extracts.

### 2.3. Effects of Treatment on Morphological Traits of Buckwheat Husk

**Figure 4:**
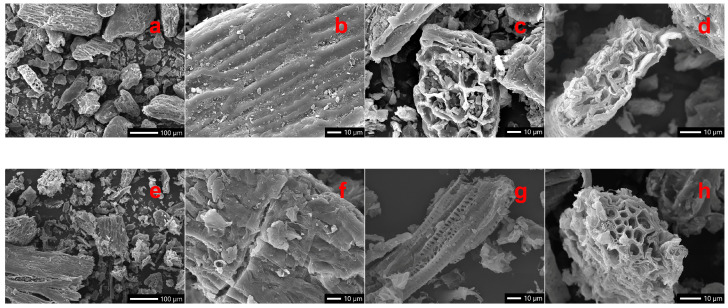
SEM micrographs of buckwheat husk before (**a**–**d**) and after microwave-assisted extraction second step MAE2 (**e**–**h**).

To explore the possibility to apply a circular economy approach for the recovery of specific compounds that may be used as a starting material for food and/or non-food applications while maximizing efficiency and sustainability, we investigated some properties of the residual solid material. The SEM images in Figure 4 show the morphological characteristics of buckwheat husk before and after MAE2, which represents the most effective treatment in promoting morphological changes in buckwheat husk. A marked increase in material fragmentation ensues under microwave treatment. In the untreated husk, cellulose fibrils appear intact and distinctly visible, exhibiting an organized structure (Figure 4a–d). Particulate matter appears with an irregular, scaly shape and forms obvious agglomerates, as also reported by Cherkashina et al. [28]. In contrast, after microwave processing, fibrils appear to be broken and disrupted, indicating significant degradation of the lignocellulosic matrix (Figure 4e–h). This morphological transformation highlights the efficacy of microwave-assisted extraction in breaking down the lignocellulosic matrix, with concomitant exposure of cellulose fibrils.

**Figure 5:**
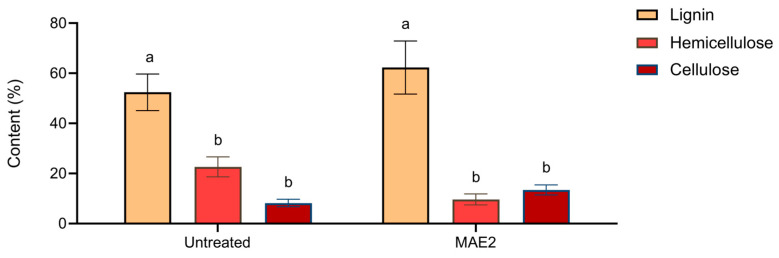
Van Soest analysis. Composition of the buckwheat husk before (untreated) and after microwave-assisted extraction second step (MAE2). Results are presented as means ± SD (*n* = 2). Data marked with the same letter are not significantly different (*p* < 0.05, Tukey’s test).

These observations are consistent with the Van Soest analysis (Figure 5), which indicates a decrease in hemicellulose content with a concomitant (relative) increase in cellulose. Such structural modifications are also responsible for improving the release of phenolic compounds, as made evident by TPC data in Table 1.

**Figure 6:**
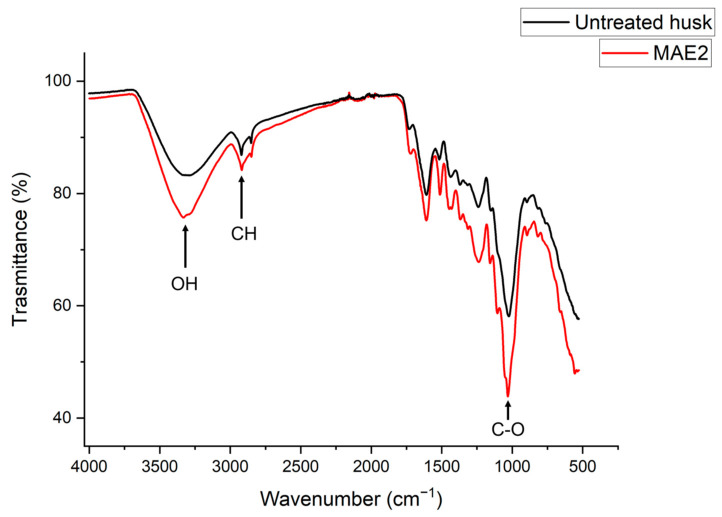
FTIR analysis of the before (untreated) and after (MAE2) microwave-assisted extraction of the buckwheat husk.

Changes in overall polysaccharide structure occurring in buckwheat husk following MAE were also investigated by FTIR spectroscopy (Figure 6). Notable differences were observed between untreated and treated samples, particularly in the intensity and shape of absorption bands in the appropriate fingerprint region (1500–500 cm^−1^). After MAE treatment, a decrease in the intensity of characteristic peaks associated with polysaccharides and lignin structures—such as those around 1030 cm^−1^ (C–O stretching vibrations) and 1230–1270 cm^−1^ (lignin-related vibrations)—was evident. The intensity decrease in these spectra also indicates that the microwave treatment breaks down hydrogen bonds, leading to a disruption of polysaccharides–lignin interactions. The spectra in Figure 6 also show shifts—and a decreased overall intensity—in the broad –OH stretching band around 3300 cm^−1^ and C–H stretching bands near 2900 cm^−1^, suggesting partial degradation of hemicellulose and structural rearrangements in the lignocellulosic matrix. These spectral modifications corroborate the SEM and Van Soest analysis data, indicating that microwave-assisted extraction significantly alters the structural and chemical profile of the husk, resulting in an enhanced accessibility of polyphenols to the solvent as indicated by the TPC yields reported in Table 1.

## 3. Discussion

In this study, we investigate the potential of two “green” approaches—namely, MAE and UAE—for the extraction of polyphenolic compounds, addressing their efficacy and their ability to overcome entrapment by the lignocellulosic matrix in buckwheat husks. MAE achieved the highest extraction efficiency, particularly when performing a second extraction step, with yields comparable to those obtained by using the harsh chemical conditions associated with the conventional Met-HCl extraction.

MAE and UAE are both able to disrupt the lignocellulosic structure of plant matrices, albeit through distinct mechanisms. UAE relies on acoustic cavitation produced by ultrasound wave propagation through the extraction solution [25,29], whereas MAE utilizes electromagnetic radiation to induce dielectric heating in the target material, resulting in cell membrane rupture and release of intracellular contents into the medium [30]. Multiple studies have highlighted the superior efficacy of MAE over UAE for extraction of phenolics from several plant matrices. For instance, Antón et al. [26] demonstrated that, from partially defatted chia flour, MAE yielded 1425.20 mg GAE/100 g in just 3.5 min compared to 871.26 mg GAE/100 g after 20 min when using UAE. Similarly, Álvarez-Romero et al. [31] reported enhanced polyphenolic compound recovery from various barley varieties using MAE, with yield increases ranging between 25% and 32% with respect to UAE, and a higher antioxidant activity. Amancio de Jesus et al. [32] extracted phenolic compounds from *Lantana camara* Linn. leaves, and found that MAE gave a slightly higher total phenolic content than UAE, but again with higher antioxidant activity (in both DPPH and ABTS assays) in MAE extracts.

Our study confirms a lower antioxidant activity in UAE extracts compared to both MAE1 and MAE2, in line with the studies commented above. Our morphological studies indicate that the enhanced performance of MAE can be attributed to the ability of microwave energy to disrupt the lignocellulosic matrix in buckwheat husks, generating internal pressure and leading to structural breakdown of the polymeric matrix. This results in more efficient heat and mass transfer, facilitating the solubilization of phenolics. In contrast to MAE, UAE acts from the exterior to the interior, with a modest effect on matrix structural compactness and on solute diffusion and solubilization [33].

Nonetheless, some studies have reported higher extraction yields with UAE than using MAE. UAE from dried *Myrtus communis* achieved a polyphenol yield of 241.60 ± 12.77 mg GAE/g, significantly surpassing the 119.59 ± 8.40 mg GAE/g obtained via MAE [22]. Also, a study by Baltacıoğlu et al. [23] found UAE to be more effective than MAE for extracting phenolic compounds from peach pomace, with a yield of 45.13 ± 1.09 mg GAE/100 g FW, compared to 34.40 mg GAE/100 g FW obtained by MAE. Finally, Duque-Soto et al. [24] observed that UAE consistently outperformed MAE, with polyphenol yields reaching 233.20 mg GAE/g extract from *Hibiscus* leaves. These discrepancies highlight the influence of multiple factors on extraction efficiency, with a prominent role played by the plant matrix composition and structure, by the operational parameters, and by the size, nature, and chemical status of specific target compounds [34,35].

The principal flavonoid identified in buckwheat husk extracts was rutin, detected consistently across all extracts. Additionally, peaks corresponding to catechin and epicatechin were observed in various extracts, as reported by Lee et al. [36]. However, quercetin was not observed in our samples, consistent with previous studies [36,37]. Regarding phenolic acids, ferulic acid was present in all samples. Confirming what was reported by Park et al. [38], our study also found that flavonoids are generally better extracted by organic solvents than by water. This trend is evident when comparing Met-HCl and BA samples. However, MAE samples showed comparable extraction of catechin and epicatechin compounds. For rutin, the trend was held across all samples, with the highest peak observed in the methanolic extract.

The antioxidant capacity of the extracts was evaluated using both ABTS and DPPH radical inhibition assays. Results indicated that MAE2 extracts exhibited the highest antioxidant potential, followed by Met-HCl and MAE1 extracts. Except for UAE, green extracts displayed distinct polyphenol profiles (Figure 1), which may account for differences in antioxidant activity. The ABTS and DPPH assays yielded different results for the green extracts, but not for the methanol extract. Specifically, the antioxidant activity measured by DPPH was lower than that assessed by the ABTS assay. This discrepancy can be attributed to the DPPH assay’s tendency to underestimate the antioxidant activity of dihydrochalcone- and flavanone-rich extracts, as noted by Platzer et al. [27]. To further investigate this, additional analyses, such as LC-MS, are suggested to identify the unknown peaks in the extracts. These findings suggest that while the absolute TPC may vary among extraction methods, the relative antioxidant capacity per unit of phenolic content remains quite similar across different extraction techniques. This observation underscores the importance of considering both the quantity of phenolic compounds extracted and their corresponding antioxidant activity when evaluating the efficacy of various extraction methodologies.

The biological activity of the extracts evaluated in Caco-2 cells indicated that MAE1 and MAE2 showed the highest viability values, comparable to those of the conventional Met-HCl extract, confirming the biocompatibility of the extracts within the tested range. Finally, our anti-inflammatory data suggests that the phenolic compounds extracted through both green and conventional methods retain the biological activities relevant to inflammation modulation, as observed in previous studies.

## 4. Materials and Methods

### 4.1. Buckwheat Materials

Buckwheat husk was provided by Molino Filippini s.r.l., Teglio (SO), Italy, in 2022. Buckwheat husk was ground by using a laboratory mill (IKA A11 basic, IKA-Werke GmbH & Co. KG, Staufen, Germany) before performing the analysis. All the chemicals and analytical standards (methanol, chloric acid, glacial acetic acid, ABTS, Folin reagent, acetonitrile, gallic acid, Trolox, TFA, catechin, epicatechin, quercitin, rutin, and ferulic acid) were from Sigma-Aldrich (St. Louis, MO, USA).

### 4.2. Extraction

#### 4.2.1. Acidified Methanol Extraction

The polyphenolic fraction was extracted as described by Lingua et al. [39], with slight modifications. Approximately 0.1 g of buckwheat husk was suspended in 2 mL of acidified methanol and stirred for 2 h in the dark. After 2 h, the tube was centrifuged at 5000× *g* for 10 min. The supernatant was collected, and the pellet was resuspended in another 2 mL of acidified methanol; the extraction was then repeated. After centrifugation, the second supernatant was combined with that obtained from the first extraction. The combined supernatants were stored at −20 °C until analysis and labeled as Met-HCl.

#### 4.2.2. Ultrasound-Assisted Extraction

Ultrasound-assisted extraction was performed in acidified water as described by Dzaha et al. [40], with some modifications. In a glass tube with a conical bottom, 0.1 g of ground buckwheat husk was suspended in 4 mL of water acidified with 0.25% (*v*/*v*) glacial acetic acid. The suspension underwent 15 sonication cycles of 1 min each, with a 1 min interval between cycles to prevent overheating, using a Soniprep 150 (MSE Ltd, London, UK) at a frequency of 16 Hz. The mixture was then transferred to a polypropylene tube and centrifuged at 5000× *g* for 15 min. The resulting supernatant was collected and labeled as UAE.

#### 4.2.3. Microwave-Assisted Extraction [41]

Microwave-assisted extraction was performed using a laboratory microwave reactor (Multiwave PRO, Anton Paar, Graz, Austria) equipped with two 1000 W magnetrons (maximum output power: 1800 W). The sample (8 g in 320 mL of water acidified with 0.25% *v*/*v* glacial acetic acid) was distributed into 16 Teflon reaction vessels (20.5 mL each). Prior to heating, samples were homogenized in an Ultra-Turrax for 1 min at a speed of 4. The microwave-assisted extraction procedure consisted of two steps. In the first step, samples were heated for 20 min (10 min ramp and 10 min hold) with a maximum pressure increase of 0.5 bar/s, a maximum pressure of 40 bar, a maximum power of 1500 W, and a temperature limit of 240 °C (IR 210 °C). After heating, samples were transferred to tubes and centrifuged at 4500× *g* for 10 min. The resulting supernatant was collected and designated as MAE1. The pellet was then resuspended in 20 mL of water, and the homogenization and heating steps were repeated. In the second step, heating involved a 3 min ramp and a 14 min hold (maximum pressure increase: 0.8 bar/s; maximum pressure: 40 bar; maximum power: 1800 W; temperature limit: 240 °C (IR 210 °C)). After centrifugation, the supernatant was recovered and labeled MAE2.

#### 4.2.4. Aqueous Acetic Acid Extraction

A control extraction was performed to estimate the contribution of the chemical process alone in isolating compounds from buckwheat husk, excluding the effects of the physical processes (UAE and MAE). Specifically, 0.1 g of buckwheat husk was suspended in 4 mL of water containing 0.25% *v*/*v* glacial acetic acid. The extraction was carried out in the dark under agitation for 3 h. After extraction, the sample was centrifuged at 5000× *g* for 10 min. The supernatant was then collected and stored at −20 °C. The extract was labeled as BA.

### 4.3. Characterization of the Extracts

#### 4.3.1. TPC

The total phenolic content was measured using the Folin–Ciocalteu reagent method with minor modifications: 0.1 mL of extract, 0.1 mL of Folin–Ciocalteu reagent, 0.1 mL of extraction solvent, and 0.7 mL of 20% (*w*/*v*) Na_2_CO_3_ were combined. The mixtures were kept in the dark for 20 min and then centrifuged for 3 min at 15,700× *g*. Absorbance was measured at 734 nm using a Perkin-Elmer Lambda 2 UV/VIS Spectrometer (PerkinElmer Inc., Waltham, MA, USA). Gallic acid was used as a standard. Results are expressed as milligrams of gallic acid equivalents per gram of sample (mg GAE/g).

#### 4.3.2. RP-HPLC

The extracts were characterized by RP-HPLC. The HPLC system was composed by two Waters 510 HPLC pump connected to a Waters 996 Photodiode Array detector and a Waters 717 plus Autosampler (Waters, Mildford, MA, USA). Compound separation was performed using a Symmetry column (Symmetry® C18, 5 µm, 4.6 × 250 mm, Waters, Mildford, MA, USA) with UV detection. The flow rate was set at 0.8 mL/min, and the injection volume was 0.03 mL. Elution was achieved using a linear gradient of solvent A (water with 0.1% TFA) and solvent B (acetonitrile with 0.1% TFA) as follows: 4 min isocratic 100% A, from 0% B to 45% B in 45 min; 4 min isocratic 100% B (the column was subsequently re-conditioned to 100% A). Phenolic acids were detected at 280 nm and 350 nm. Standards of ferulic acid, rutin, quercetin, catechin, and epicatechin were used to identify the phenolic compounds present in the various extracts obtained.

### 4.4. Antioxidant Activity Assays

#### 4.4.1. ABTS Assay

The antioxidant capacity was assessed using the ABTS radical inhibition method, as described by Re et al. [42]. Briefly, 0.01 mL of sample was added to 0.990 mL of ABTS solution, and the absorbance was measured at 734 nm in a Perkin-Elmer Lambda 2 UV/VIS Spectrometer (PerkinElmer Inc., Waltham, MA, USA) after 1 min. Trolox was used to generate a calibration curve, and results were expressed as mmol of Trolox equivalents per kilogram of sample (mmol TE/kg). The results were normalized to the total phenolic content (TPC) determined by the Folin–Ciocalteu assay. From each extract, at least two aliquots were taken to measure the antioxidant activity.

#### 4.4.2. DPPH Assay

Antioxidant capacity was assessed using the DPPH radical inhibition method as reported by Valli et al. [43]. The Trolox standard was prepared in ethanol at concentrations ranging from 0 to 2.5 mM. A volume of 0.01 mL of each standard or sample was added to 2.9 mL of a 0.1 mM DPPH solution in methanol/water (80:20 *v*/*v*). Absorbance was measured at 517 nm in time-drive mode for 30 min. Results were expressed as mmol of Trolox equivalents per kilogram of sample (mmol TE/kg). The results were normalized to the total phenolic content (TPC) as determined by the Folin–Ciocalteu assay. From each extract, at least two aliquots were taken to measure the antioxidant activity.

### 4.5. Caco-2 Cell Cultivation

Human intestinal epithelial Caco-2 cells were cultured in 75 cm^2^ flasks using low-glucose Dulbecco’s Modified Eagle’s Medium (DMEM), supplemented with 10% (*v*/*v*) heat-inactivated fetal bovine serum (FBS), penicillin (100 U/mL), streptomycin (0.1 mg/mL), and L-glutamine (2 mM). The cells were maintained in a humidified atmosphere of 5% CO_2_ at 37 °C, with medium changes every 2–3 days. Once the cells reached confluence, they were transferred into multi-well plates for subsequent experiments.

### 4.6. MTT Viability Assay

Caco-2 cells were seeded at a density of 0.5 × 10^5^ cells/mL in a 24-well plate and incubated for 48 h to allow for cell adhesion and growth. The cells were then exposed to bioactive extracts from buckwheat husks at concentrations of 6.25 mg/L, 25 mg/L, and 50 mg/L, alongside a vehicle control group. After 24 h of treatment, 0.05 mL of 3-(4,5-Dimethylthiazol-2-yl)-2,5-diphenyltetrazolium bromide (MTT) solution (3 mg/mL) was added to each well and the plate was incubated for 3 h at 37 °C to facilitate formazan formation [44]. Subsequently, the medium was removed, and 0.5 mL of dimethyl sulfoxide was added to each well to dissolve the formazan. The plate was gently agitated for 10 min in the dark to ensure complete solubilization. From each well, 0.15 mL of the solubilized formazan solution was transferred in duplicate to a 96-well plate for absorbance measurement. The optical density was then measured at 565 nm using Infinite^®^ M Nano^+^ (TECAN group Ltd, Männedorf, Switzerland) Multiple Plate Reader. The cell viability was calculated by comparing the absorbance of treated cells to that of the control group, which was set as 100% viability.

### 4.7. Anti-Inflammatory Assay

#### 4.7.1. Cell Incubation

Caco-2 cells were seeded at a density of 1 × 10^5^ cells/mL in a 24-well plate and incubated for 48 h to allow for cell adhesion and growth. The cells were then stimulated with IL-1β (20 ng/mL) in the presence or absence of buckwheat extracts to assess their potential anti-inflammatory properties. Three concentrations of buckwheat extracts were tested—6.25 mg/L, 25 mg/L, and 50 mg/L—alongside a vehicle control group. Cells treated with IL-1β alone served as positive control, establishing 100% inflammation induction, while untreated cells were used as the negative control. The effects of buckwheat extracts on inflammation were evaluated by analyzing changes in the expression of target genes relative to the untreated control [45]. All treatments were performed in duplicate for each sample.

#### 4.7.2. RNA Extraction

After 1 h of treatment, the total RNA was extracted using the Aurum Total RNA Kit (7326820, Bio-Rad Laboratories Inc., Hercules, CA, USA) following the manufacturer’s protocol. The cells were lysed with lysis solution containing 1% (*v*/*v*) β-mercaptoethanol. The lysates were then transferred to RNA binding columns, mixed with 70% (*v*/*v*) ethanol/water, and centrifuged at 13,400× *g* for 30 s. RNA purification was performed through sequential rinses with low- and high-stringency wash solutions, each followed by centrifugation at 13,400× *g* for 30–60 s. DNAse I digestion was carried out to remove genomic DNA contamination. Finally, the RNA was eluted with 0.08 mL of elution solution, and the eluate was stored at −20 °C or −80 °C until further processing.

#### 4.7.3. Reverse Transcription PCR (RT-PCR)

Total RNA was reverse-transcribed into complementary DNA (cDNA) using the iScript Reverse Transcription Supermix for RT-qPCR kit (1708841, Bio-Rad Laboratories Inc., Hercules, CA, USA) with 0.005 mL of an RNA sample, resulting in a final volume of 0.02 mL for the reaction mixture. The reverse transcription reaction was carried out in a Mastercycler Personal 5332 (Eppendorf AG 22331, Hamburg, Germany) using the pre-set program under the following conditions: 5 min at 25 °C, 20 min at 46 °C, 1 min at 95 °C to terminate the reaction, and 4 °C for 4 h to stabilize the cDNA. The resulting cDNA was subsequently stored at −20 °C or −80 °C for future use.

#### 4.7.4. Expression of IL-8 Measurement by Real-Time PCR Analysis (qPCR)

Gene expressions of IL-8 and the housekeeping gene GAPDH were quantified using qPCR on a CFX Connect Real-Time PCR Detection System (Bio-Rad Laboratories Inc., Hercules, CA USA). Reactions were performed in a final volume of 0.020 mL, containing 0.002 mL of cDNA, 0.01 mL of SsoAdvanced Universal SYBR Green Supermix (1725271, Bio-Rad Laboratories Inc., Hercules, CA, USA), and 0.004 mL of each primer (0.003 mM). The primers used for IL-8 amplification were [46] IL-8 (Forward): 5′-CTGGCCGTG…TTCCTG-3′ (00270901-3, Biomers, Ulm, Germany); IL-8 (Reverse): 3′-GGCAACCCTA…AATACA-5′ (00270901-4, Biomers). The GAPDH gene was amplified using the following primers [47]: GAPDH (Forward): 5′-GGAAGGTGAA…GGAGTC-3′ (00270901-1, Biomers); GAPDH (Reverse): 5′-CACAAGCTTC…TCTCAG-3′ (00270901-2, Biomers). Thermal cycling conditions consisted of an initial denaturation at 95 °C for 5 min, followed by 40 cycles of denaturation at 95 °C for 20 s, annealing at 62 °C for 30 s, and extension at 72 °C for 30 s. Relative gene expression levels were calculated using the Livak method [48], with GAPDH as the reference gene. Each sample was analyzed in duplicate.

### 4.8. SEM, FTIR and Van Soest Analysis

#### 4.8.1. SEM

The samples were analyzed after gold metallization using a Sacncoat Six Sputter Coater by Edwards (Burgess Hill, UK). The images were taken under high vacuum (HV) conditions, using an accelerating voltage of 20 kV, a probe current of 2.54 μA, and a beam current (P.C.) of 40 μA. The equipment used in the laboratory is a SEM-EDS JSM-IT500 LA by JEOL Spa (Basiglio, Milan, Italy).

#### 4.8.2. FTIR

The analysis was conducted using a Thermo Fisher Nicolet iS50 FT-IR spectrometer equipped with a SMART iTX single-bounce ATR accessory (Thermo Scientific, Waltham, MA, USA). Spectral data were acquired with OMNIC 9.14.97 software, which provides automatic accessory recognition and parameter setup to ensure optimal data collection. The sample was placed directly onto the crystal plate of the ATR accessory, and spectra were recorded in the range of 4000 to 500 cm^−1^ with a resolution of 4 cm^−1^.

#### 4.8.3. Van Soest

Van Soest analyses were carried out by Innovhub (Stazioni Sperimentali per l’Industria S.r.l., Milan, via G. Colombo 79).

### 4.9. Statistical Analysis

Statistical analysis was performed using GraphPad Prism version 10.0 (GraphPad Software, San Diego, CA, USA). Data are expressed as mean ± standard deviation (SD). Statistical significance was assessed using one-way ANOVA, followed by Tukey’s post hoc test for multiple comparisons. *p*-values < 0.05 were considered statistically significant.

## 5. Conclusions

This study demonstrates the potential of green extraction techniques, particularly microwave-assisted extraction, to valorize buckwheat husk as a sustainable source of bioactive polyphenolic compounds. Both microwave-assisted extraction and ultrasound-assisted extraction, using water-based solvents, were effective in extracting phenolics while preserving their antioxidant capacity. Microwave-assisted extraction exhibited the highest extraction efficiency: the two extraction steps (MAE1 and MAE2) together provided an extraction yield more than double compared to conventional extraction with acidified methanol.

In addition to favorable chemical characteristics, the extracts showed no cytotoxic effects and demonstrated anti-inflammatory activity in an intestinal epithelial model, confirming their biological relevance. These findings support the application of buckwheat husk extracts in food systems, particularly as natural preservatives or shelf-life extenders, due to their antioxidant potential.

MAE treatments induced also a significant degradation of the lignocellulosic matrix, with concomitant exposure of cellulose fibrils. The materials obtained may be more suitable for biobased material production.

The overall data reported in this study suggest a valorization of buckwheat husk in various applications, acting in a contest of sustainability and circular economy.

## Figures and Tables

**Table 1:** Total phenolic content (TPC) and antioxidant activity (ABTS and DPPH) of buckwheat husk extracts.

Extraction Method	TPC (mg GAE/g)	ABTS (mmol TE/g GAE)	DPPH (mmol TE/g GAE)
Met-HCl	9.68 ± 0.13 ^b^	9.00 ± 0.02 ^b^	8.34 ± 0.05 ^a^
BA	1.00 ± 0.01 ^d^	8.01 ± 0.25 ^c^	5.31 ± 0.04 ^c^
UAE	1.11 ± 0.02 ^d^	8.02 ± 0.08 ^c^	6.38 ± 0.03 ^b^
MAE1	7.15 ± 0.05 ^c^	9.52 ± 0.17 ^a^	4.90 ± 0.21 ^c^
MAE2	13.90 ± 0.09 ^a^	9.14 ± 0.02 ^b^	7.56 ± 0.63 ^a^

Results are presented as means ± SD (*n* = 2). Different superscript letters in one column indicate significant differences (*p* < 0.05, Tukey’s test). Met-HCl, acidified methanol extracts; BA, Aqueous Acetic Acid Extract; UAE, ultrasound-assisted extraction; MAE1, microwave-assisted extraction first step; MAE2, microwave-assisted extraction second step; TPC, total phenolic content expressed as mg GAE/g of dry sample. ABTS and DPPH: antioxidant activity was measured by ABTS and DPPH assays, both normalized to TPC and expressed as mmol Trolox equivalents (TE) per g of GAE.

## Data Availability

Dataset available on request from the authors.
